# ﻿Review of the genus *Gigantothrips* Zimmermann from China and Southeast Asia (Thysanoptera, Phlaeothripidae, Phlaeothripinae)

**DOI:** 10.3897/zookeys.1169.106733

**Published:** 2023-07-14

**Authors:** Lihong Dang, Laurence Mound, Hongrui Zhang

**Affiliations:** 1 School of Bioscience and Engineering, Shaanxi University of Technology, Hanzhong, 723000, China Shaanxi University of Technology Hanzhong China; 2 Shaanxi Province Key Laboratory of Bioresources, Hanzhong, 723000, China Shaanxi Province Key Laboratory of Bioresources Hanzhong China; 3 Qinba Mountain Area Collaborative Innovation Center of Bioresources Comprehensive Development, Hanzhong, 723000, China Qinba Mountain Area Collaborative Innovation Center of Bioresources Comprehensive Development Hanzhong China; 4 Qinba State Key Laboratory of Biological Resources and Ecological Environment (Incubation), Hanzhong, 723000, China Qinba State Key Laboratory of Biological Resources and Ecological Environment Hanzhong China; 5 Australian National Insect Collection CSIRO, PO Box 1700, Canberra, ACT 2601, Australia Australian National Insect Collection Canberra Australia; 6 Plant Protection College, Yunnan Agricultural University, Kunming, 650201, China Yunnan Agricultural University Kunming China

**Keywords:** Identification key, leaf-feeding, *Liothrips*-lineage, new species, taxonomy, thrips

## Abstract

*Gigantothrips* is a genus of leaf-feeding species from the Old World tropics that is distinguished from *Gynaikothrips* and *Leeuwenia* by the large number of tergal wing-retaining setae. Eight species are recognized from China and Southeast Asia including *G.tibetanus***sp. nov.** from Tibet and *G.yunnanensis***sp. nov.** from Yunnan, both taken on the leaves of *Ficus* trees. An illustrated identification key to these eight species is provided here.

## ﻿Introduction

Species of the subfamily Phlaeothripinae exhibit the widest range of feeding habits in the order Thysanoptera, being known as plant-living, fungus-feeding and predators. These feeding habits largely accord with the three informal groups recognized (Mound and Marullo 1996). The ‘*Haplothrips*-lineage’ comprises primarily flower-living species, and is now considered a formal taxon, the tribe Haplothripini (Mound and Minaei 2007). The ‘*Phlaeothrips*-lineage’ includes mainly fungus-feeding species that are found in dry or dead leaves and branches, leaf litter and dry grasses. The third group, the ‘*Liothrips*-lineage’, is one of the most species-rich groups in Thysanoptera, with most species feeding on green leaves of trees or shrubs, and often associated with leaf rolls or galls (Mound 1971; Ananthakrishnan 1972; Mound 1994; Mound 2020a, b; Mound and Tree 2021a, b). Found in tropic or subtropic regions of the Old World, species of the genus *Gigantothrips* Zimmermann are associated with leaf-galls on *Ficus* trees. This genus is closely related to *Gynaikothrips* Zimmermann and *Leeuwenia* Karny (Mound and Tree 2021a). *Gigantothrips* was erected very early in 1900, and is one of the ‘oldest’ genera in Phlaeothripinae – only nine other genera were established in 1900 or before (Zimmermann 1900; ThripsWiki 2023). However, only a few publications were concerned with this genus and only two keys are currently available. The latest one was produced 43 years ago, in which six species were recognized from India (Ananthakrishnan and Sen 1980). Before that a key to nine species, including six from Africa based on material in the Natural History Museum, London, was produced by Mound (1968). A little further information on *Gigantothrips* was mentioned for Chinese and Southeast Asian species (Dang et al. 2014; Mound and Tree 2021b). Here we present a review of *Gigantothrips* with a key to eight species from China and Southeast Asia.

*Gigantothrips* shares with *Gynaikothrips* and *Leeuwenia*, amongst other members of *Liothrips*-lineage, the following character states, metathoracic sternopleural sutures absent, mesopresternum boat-shaped, pronotum with irregular reticulate sculpture. Currently, *Gigantothrips* is differentiated from *Leeuwenia* in having the tube relatively short compared to the large body, major wing-retaining setae on tergites not fan-shaped, and the head parallel-sided with weak sculpture (Mound 2004); and from *Gynaikothrips* in the large body size (about 5 cm), numerous sigmoid or straight wing-retaining setae on tergites II–V, and long tube bearing fine setae on the surface (Dang et al. 2014). Based on the above, two new species from Yunnan and Tibet of China are here recognized as members of *Gigantothrips*.

## ﻿Material and method

The descriptions, photomicrograph images and drawings were produced from slide-mounted specimens with a Nikon Eclipse 80i microscope. Images were prepared with a Leica DM2500 using differential interference contrast (DIC) illumination, and processed with Automontage and Photoshop v.7.0. The abbreviations used for the pronotal setae are as follows: **am** – anteromarginal, **aa** – anteroangular, **ml** – midlateral, **epim** – epimeral, **pa** – posteroangular. The unit of measurement in this study is the micrometre. Most specimens studied here are available in the Australian National Insect Collection (**ANIC**), Canberra, Australia, the School of Bioscience and Engineering, Shaanxi University of Technology (**SUT**), Hanzhong, China, and the National Zoological Museum of China (**NZMC**), Institute of Zoology, Chinese Academy of Sciences, Beijing, China. Additionally, three slides were on loan from the Naturmuseum Senckenberg (**SMF**), Frankfurt, Germany.

## ﻿Taxonomy

### ﻿ Gigantothrips

Taxon classificationAnimalia

Zimmermann, 1900

9AC99836-0675-5270-BDC2-1226625C97DD

Gigantothrips Zimmermann, 1900: 18. Type species Gigantothripselegans Zimmermann, 1900, by monotypy.

#### Note.

There are now 22 species listed in this genus (ThripsWiki 2023), of which 10 are from Africa, one from Mexico, and five from India. The record from Mexico is likely to be due to mislabeling, as the three known specimens apparently represent an Asian species (Mound and Marullo 1996). The genus is thus considered to be entirely from the Old World tropics. *Gigantothripselegans* Zimmermann is widespread from India to the Philippines, including southern China, on the leaves of *Ficus*. *G.tibialis* Bagnall was described from Sri Lanka but later recorded from Hainan, China (Zhang 1984), *G.nigrodentatus* (Karny) was described from Java and then recorded from India, whereas *G.gallicola* (Priesner) is known only from Java. Two species only from the Philippines, *G.pontis* (Reyes) and *G.xynos* (Reyes), were transferred to this genus from *Gynaikothrips* by Dang et al. (2014). Now there are eight species recognized from China and Southeast Asia including two new species found in Tibet and Yunnan, *G.tibetanus* sp. nov. and *G.yunnanensis* sp. nov.

#### Diagnosis.

Head much longer than wide, cheeks parallel-sided with a few stout setae (Figs 1–3, 7–10); eyes normal, postocular setae usually undeveloped; stylets retracted to one third of head, close together; antennae slender, 8-segmented, III with 1 sense cone, IV with 3, rarely 2 (Figs 15–17); pronotum with major setae often short, notopleural sutures incomplete (or complete) (Figs 1–3, 11–14); basantra absent; mesopresternum boat-shaped; sternopleural sutures absent; fore tarsal tooth usually present in both sexes; fore wings parallel-sided, with numerous duplicated cilia; pelta triangular (Figs 18, 21, 22); tergites II–VI with numerous sigmoid or straight wing-retaining setae (Figs 4, 23–25); tube long and slender with fine setae on the surface, usually longer than head, anal setae short (Figs 5, 6, 32, 33); male sternite VIII with or without pore plate (Figs 28–30).

##### ﻿Key to *Gigantothrips* species from China and Southeast Asia

**Table:** 

1	All tibiae clearly yellow	**2**
–	At least mid and hind tibiae brown at base	**3**
2	Pronotal aa close together with midlateral setae, the distance between them shorter than their length (Fig. 1) (on *Careyaarborea*)	***Gigantothripstibialis* Bagnall**
–	Pronotal aa far away from midlateral setae, the distance between them longer than their length (Fig. 3) (on *Ficuspseudopalma*)	***G.pontis* (Reyes)**
3	Tube short, no more than five times as long as basal width; S1–S2 on tergite IX acute and slightly shorter than tube (Fig. 6) (on *Euphobiahirta*)	***G.xynos* (Reyes)**
–	Tube elongate, more than eight times as long as basal width; S1–S2 on tergite IX blunt and much shorter than tube (Fig. 5)	**4**
4	Anterior margin of pronotum with about eight pairs of stout setae (Fig. 12) (on *Ficus* spp.)	***G.elegans* Zimmermann**
–	Anterior margin of pronotum with four pairs of setae at most	**5**
5	Fore tibiae clear yellow	**6**
–	Fore tibiae largely brown or shaded with brown	**7**
6	Antennal segment IV with 3 sense cones; major setae stout and hyaline (Fig. 7); tergites II–V with at least six sigmoid wing-retaining setae (Fig. 23); pronotal pa minute, much smaller than aa (Fig. 11) (on *Planchoniavalida*)	***G.nigrodentatus* Karny**
–	Antennal segment IV with 2 sense cones (Fig. 17); major setae slender and yellow or brown; tergites II–V with two major sigmoid wing-retaining setae (Fig. 8); pronotal pa as long as aa (on leaves of *Ficustikoua*)	***G.yunnanensis* Dang & Mound, sp. nov.**
7	Tergites II–VI with 3–4 pairs of major sigmoid setae, and numerous small sigmoid setae laterally; cheek with about 8 pairs of short and acute, but spinous setae (Fig. 2); antennal segment III uniformly yellow, IV–VI yellow with lightly brown at apical, VII bicolored with basal half yellow, VIII brown (in leaf-galls of *Sloanea* sp.)	***G.gallicola* Priesner**
–	Tergites II–VI with two pairs of major sigmoid setae, and numerous straight setae laterally (Fig. 24); cheek with about 8 pairs of short and acute, but slender setae (Fig. 10); antennal segments III–VI yellow with shaded at apical, VII–VIII uniformly brown (on leaves of *Ficus* sp.)	***G.tibetanus* Dang & Mound, sp. nov.**

**Figures 1–6.**
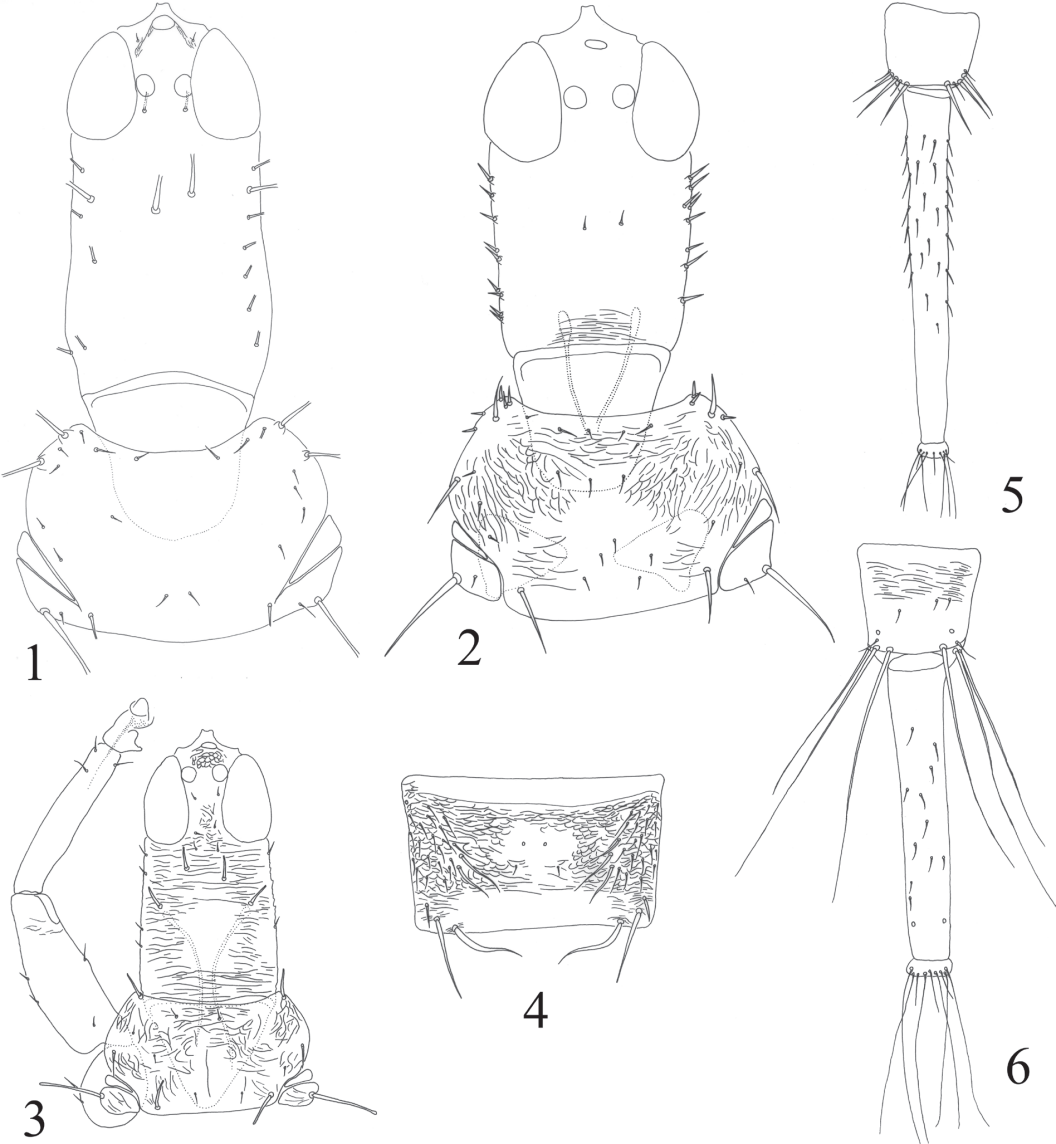
*Gigantothrips* spp. Head and pronotum (**1, 2**) **1***G.tibialis***2***G.gallicola*; head, pronotum and fore leg (**3**) **3***G.pontis*; tergite IV (**4**) **4***G.xynos*; tergites IX–X **(5, 6) 5***G.gallicola***6***G.xynos*.

### ﻿ Gigantothrips elegans

Taxon classificationAnimalia

Zimmermann

771EABDC-916C-5392-BC04-568704864331

Figs 9 , 12 , 15 , 18 , 25

Gigantothrips elegans Zimmermann, 1900: 18.

#### Material examined.

20♀3♂(NZMC), China, Hainan, 07.iv.1958, X.L. Meng; 1♀ (NZMC), Hainan, 18.v.1983, M.S. Shuo; 2♂ (NZMC), Hainan, 20.v.1985, M.S. Shuo; 1♀ (NZMC), Hainan, 20.v.1963, B.L. Zhang.

#### Comments.

Described from Java, Indonesia, this species is widespread in the tropical area from India to the Philippines, including southern China, feeding on leaves of *Ficus* species (Bagnall 1908; Hood 1919; Sen, Pramanik and Sengupta 1988). This species differs from other *Gigantothrips* species in having numerous remarkable stout setae on the anterior margin of the pronotum (Fig. 12). Studied here were 22 females and five males from Hainan Island, and they have at least five pairs of sigmoid retaining setae on tergites II–V although there are no wing-retaining setae on VI–VII (Fig. 25).

### ﻿ Gigantothrips gallicola

Taxon classificationAnimalia

(Priesner)

7BD16504-9389-59B2-B15C-DBFD17E86F6B

Figs 2 , 5

Syringothrips gallicola Priesner, 1933: 77.

#### Material examined.

***Holotype***, 1♀ (SMF), Indonesia, Java, in leaf-gall of *Sloanea* sp., 26.iii.1925.

#### Comments.

Described from Java, Indonesia, this species represented a new genus, ‘*Syringothrips*’, based on only a female originally. After checking the holotype female (on loan from the Senckenberg Museum, Frankfurt), it was considered to be a member of *Gigantothrips* and related to *G.nigrodentatus* from Java (Dang et al. 2014). However, *G.gallicola* was found in the leaf-galls of *Sloanea* sp. whereas *G.nigrodentatus* has been found breeding on the leaves of *Planchoniavalida*. This species is also similar in body colour and shape to *G.tibetanus* sp. nov., but they can be distinguished by means of couplet 7 in the key.

### ﻿ Gigantothrips nigrodentatus

Taxon classificationAnimalia

(Karny)

A3B3298F-B81D-59C2-BA54-FE9C549EAA9C

**Figures 7–14.**
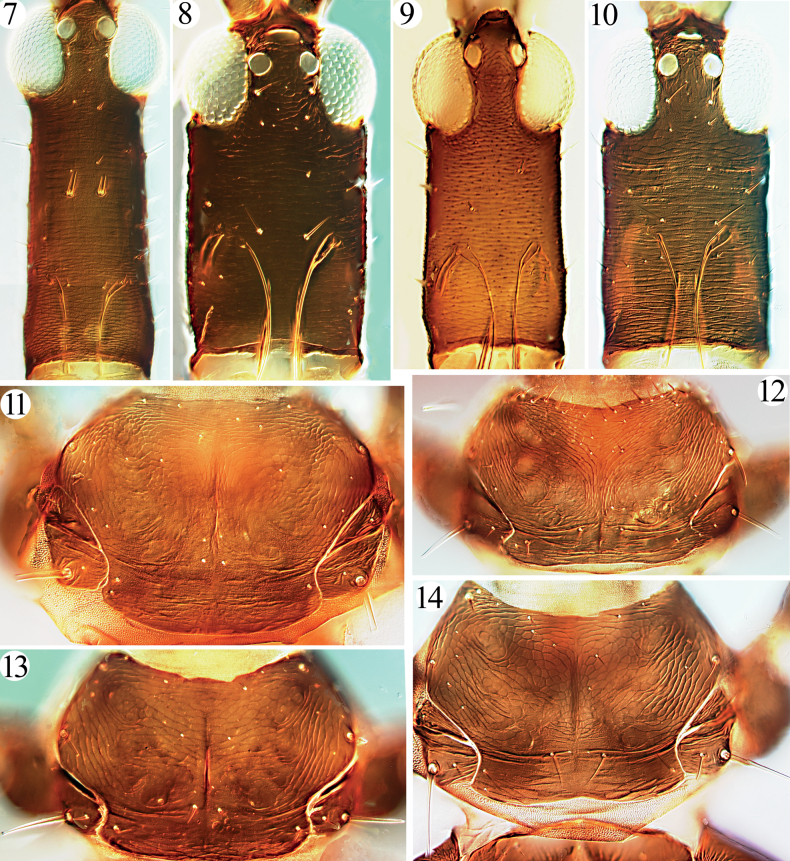
Head and pronotum. Head (**7–10**) **7***G.nigrodentatus***8***G.yunnanensis* sp. nov. **9***G.elegans***10***G.tibetanus* sp. nov.; pronotum (**11–14**) **11***G.nigrodentatus***12***G.elegans***13***G.yunnanensis* sp. nov. **14***G.tibetanus* sp. nov.

Figs 7 , 11 , 23 , 30

Acanthinothrips nigrodentatus Karny, 1913: 120.

#### Material examined.

1♀1♂ (ANIC), Indonesia, Java, on curled leaves of *Planchoniavalida*, 30.x.1973, L.A. Mound.

#### Comments.

This species was described originally from Java, Indonesia, but was considered a member of Idolothripinae within the invalid genus ‘*Cercothrips*’ because of the large size body (Hood, 1919). After comparing the type specimens, the species was identified as belonging to the Phlaeothripinae as a species of *Gigantothrips* by Mound (1968). A female and a male are studied here, taken on *Planchoniavalida* in Java by Mound in 1973. It is similar to *G.yunnanensis* sp. nov. in body sculpture and the colour pattern of legs, but they can be differentiated in the above key.

### ﻿ Gigantothrips pontis

Taxon classificationAnimalia

(Reyes)

CAD5C711-6677-5E02-8A8D-FB32E8C8D912

Figs 3 , 26

Gynaikothrips pontis Reyes, 1996: 112.

#### Material examined.

***Holotype***, 1♀(ANIC), the Philippines, Luzon, on *Ficuspseudopalma*, i.1985, C.P. Reyes. ***Paratype***, 1♂ (ANIC), with the same data as holotype.

#### Comments.

Described from Luzon, the Philippines, taken on *Ficuspseudopalma*, this species was newly combined to the genus *Gigantothrips* together with another Philippines species, *G.xynos*, based on the typical character state of the numerous pairs of tergal wing-retaining setae (Dang et al. 2014). The tube is relatively short, but is longer than head and has some fine setae. The type specimens of both of these species have numerous fine setae present on tergites VII–VIII medially and laterally (Figs 26, 27), but with no sigmoid setae. *G.pontis* is closely similar to *G.tibialis* with all tibiae clear yellow which is easy to distinguish from other *Gigantothrips* species.

### ﻿ Gigantothrips tibetanus

Taxon classificationAnimalia

Dang & Mound sp. nov.

3A45E950-0F0F-59AA-83DF-D09AAC136235

https://zoobank.org/2F74AC2D-1EB4-41CD-A902-BBA508C537D3

Figs 10 , 14 , 16 , 19 , 21 , 24 , 29

#### Material examined.

***Holotype***, ♀ (SUT), China, Tibet, Motuo County, on leaves of *Ficus* sp., 19.vii.2022, Y.Q. Li. ***Paratypes***, 1♀1♂ (SUT), with the same data as holotype; 1♀ (ANIC), with the same data as holotype.

#### Description.

**Holotype. *Female macroptera*.** Body brown. All femora brown, fore tibiae brownish yellow shaded with brown medially, mid and hind tibiae brown with yellowish brown at apical area, all tarsi yellowish brown. Antennae segments I–II brown, III yellow with shaded at apex, IV–V yellow with apices shaded brown, VI brown on apical half, yellow on basal half, VII–VIII brown. Wings strongly shaded with brown, body setae yellowish.

***Head*.** Head elongate, about 2.0 times as long as wide (Fig. 10); cheeks almost straight, each with 8–9 small and slender setae, dorsal surface weakly sculptured with transverse striae; ocelli area prominent, a pair of postocellar setae as long as postocular setae (Fig. 10); eyes about third of head length, two pairs of postocular setae small and slender, about as long as cheek setae (Fig. 10). Mouth-cone rounded, maxillary stylets rather short, not reaching the middle of head, close together. Antennal 8-segmented, intermedial segments elongate, III longer than IV, about 7.0 times as long as width, sensoria long and slender, III with 0+1, VI with 1+2 (Fig. 16).

***Thorax*.** Pronotum sculptured with irregular striae and reticulation, notopleural suture complete (Fig. 14); only epim well developed, slightly blunt at apex, am no longer than discal setae, aa slightly bigger than am with several small setae around, pa slender, no longer than other discal setae, ml as long as aa (Fig. 14); mesonotum and metanotum reticulation with internal fine markings, no developed setae on them, metanotal with four small setae around anterior angle (Fig. 19), mesopresternum boat-shaped, metathoracic sternopleural sutures absent. All legs slender, fore tarsal absent. Fore wings broad, with 27–28 duplicated cilia, sub-basal setae almost equal length, acute or slightly blunt at apex.

***Abdomen*.** Pelta triangular and reticulate with internal markings, with a pair of campaniform sensilla and 1–2 pairs of minute setae (Fig. 21); abdominal tergites II–VI with two pairs of major sigmoid wing-retaining setae, and numerous accessory setae slightly curved or straight (Fig. 24); VII with numerous small accessory setae laterally; S1–S3 on tergite IX short and pointed or slightly blunt at apex, about 0.1 times as long as tube (Fig. 32); tube elongate with fine setae on the surface (Fig. 32), 2.3 times as long as head, about 8.8 times as long as basal width, anal setae much shorter than tube; sternites II–VIII with some small setae not in lines.

***Measurements*** (holotype female in microns). Body length 5880. Head length 450, width just behind eyes 235; eye length 155, postocular setae length S1 30, S2 40; postocellar setae length 35; the narrowest separation between maxillary stylets 25. Antenna length 1025, segments I–VIII length (widest) 60(50), 60(40), 240(35), 190(45), 175(40), 150(35), 90(25) and 70(20), sensoria on segment III length 65. Pronotum length 240, width 400, length of pronotal setae, am 20, aa 25, ml 25, epim 115, pa 30. Fore wing length 1900, sub-basal setae length, S1 80, S2 70, S3 100. Pelta length 170, basal width 290; tergite IX posteromarginal setae S1–S3, 130, 135, 120; tube length 1050, basal width 120, apical width 70; anal setae length 230.

***Male macroptera*.** Similar to female; but smaller, fore tarsal tooth absent; abdominal tergite IX setae S2 short; sternite VIII with a pore plate, its anterior margin like ‘W’ shape and posterior margin straight and before posterior marginal setae (Fig. 29), sternites III–VII with a worm-shape longitudinal laterally (Fig. 31).

***Measurements*** (paratype male in microns). Body length 5420. Head length 425, width just behind eyes 205; eye length 135, postocular setae length S1 30, S2 35; postocellar setae length 30; the narrowest separation between maxillary stylets 25. Antenna length 1050, segments I–VIII length (widest) 60(50), 60(35), 240(35), 185(45), 175(40), 135(40), 85(25) and 65(20), sensoria on segment III length 75. Pronotum length 250, width 365, length of pronotal setae, am 25, aa 25, ml 30, epim 110, pa 35. Fore wing length 1850, sub-basal setae length, S1 70, S2 75, S3 80. Pelta length 150, basal width 250; tergite IX posteromarginal setae S1–S3, 130, 50, 80; tube length 980, basal width 110, apical width 60; anal setae length 230.

#### Etymology.

This species name is composed of one Latin word based on the location of type specimens.

#### Comments.

This new species was taken on the leaves of a *Ficus* species and it is quite similar in body shape and colouration to *G.gallicola* taken on the leaf-galls of *Sloanea* sp. in Java. However, *G.tibetanus* sp. nov. can be distinguished by the slender cheek setae (Fig. 10), tergites II–VI with two pairs of major sigmoid setae (Fig. 24), pronotal aa, ml and pm small and slender, as long as discal setae (Fig. 14), and metanotum with a pair of small setae located at anterior angles together with three tiny setae (Fig. 19). In *G.gallicola*, cheeks have numerous spinous setae (Fig. 2), tergites II–VI with 3–4 pairs of major sigmoid setae, pronotal aa, ml and pm developed, distinctly longer than discal setae (Fig. 2), and metanotum anterior half with a pair of small setae and four pairs of tiny setae before them, also three pairs of tiny setae located at anterior angles. The species also have different colour pattern of antennae as indicated in couplet 7 of the key.

### ﻿ Gigantothrips tibialis

Taxon classificationAnimalia

Bagnall

F19D46D1-F04B-56F8-B6E3-BF6DF6031775

Fig. 1

Gigantothrips tibialis Bagnall, 1921: 364.

#### Material examined.

1♀(SMF), India, Travancore, v.1934, S.A. Raw; 1♀(SMF), India, Mangalore, 21.i.1964, T.N. Ananthakrishnan.

#### Comments.

Described from Sri Lanka on *Careyaarborea*, this species is probably widespread in India according to Ananthakrishnan (1964: 36). However, the identifying characters mentioned by Ananthakrishnan, ‘basal third of mid and hind tibiae brown, rest yellow’, are inconsistent with the original description by Bagnall of all tibiae wholly yellow. Two females from southern India (Travancore and Mangalore) were studied here with the identical tibiae colouration given in the original description, and three major setae on the pronotum with ml close to aa (Fig. 1). This species was recorded from Hainan, China by Zhang (1984: 19), but without more identification information.

### ﻿ Gigantothrips xynos

Taxon classificationAnimalia

(Reyes)

F17FB7B6-C07C-561D-9898-5AE68380396B

Figs 4 , 6 , 27

Gynaikothrips xynos Reyes, 1996: 118.

#### Material examined.

***Holotype***, 1♀(ANIC), the Philippines, Leyte, on *Euphorbiahirta*, 14.vi.1984, C.P. Reyes. ***Paratype***, 1♀ (ANIC), with the same data as holotype.

#### Comments.

This is the third species of *Gigantothrips* recorded from the Philippines, and was taken on *Euphorbiahirta*. It has tergite IX setae S1–S3 a little shorter than the tube (Fig. 6), which is unique in comparison with other *Gigantothrips* species that have these setae much shorter than their tubes.

### ﻿ Gigantothrips yunnanensis

Taxon classificationAnimalia

Dang & Mound sp. nov.

23F0AAC2-AA45-5985-84AC-D6E033755DC2

https://zoobank.org/3219E244-9388-4C0E-ACDA-7539B38F1E3A

Figs 8 , 13 , 17 , 20 , 22 , 28 , 33

#### Material examined.

***Holotype***, ♀ (ANIC), China, Yunnan, Kunming Garden, on *Ficustikoua*, 29.ix.2010, H.R. Zhang. ***Paratypes***, 2♀(ANIC), with the same data as holotype; 1♀1♂ (ANIC), Yunnan, Kunming, Chenggong, on *Ficustikoua*, 08.viii.2010, H.R. Zhang; 1♀(SUT), with the same data as holotype.

#### Description.

**Holotype. *Female macroptera*.** Body brown. All femora brown, fore tibiae clear yellow, mid and hind tibiae yellow with brown at basal third, all tarsi yellow. Antennae segments I–II brown with paler at extremely apices, III–V uniformly yellow, VI yellow with apices shaded, VII–VIII yellowish brown. Wings very weak shaded with brown, body setae yellowish.

***Head*.** Head elongate, about 1.8 times as long as wide (Fig. 8); cheeks almost straight, each with 2–4 small setae, dorsal surface weakly sculptured with transverse striae; ocelli area prominent, a pair of postocellar setae as long as postocular setae; eyes about third of head length, two pairs of postocular setae stout and pointed at apex, longer than cheek setae (Fig. 8). Mouth-cone rounded, maxillary stylets rather short, not reaching the middle of head, close together. Antennal 8-segmented, intermedial segments elongate, III longer than IV, about 5 times as long as width, sensoria long and slender, III with 0+1, VI with 1+1 (Fig. 17).

**Figures 15–25.**
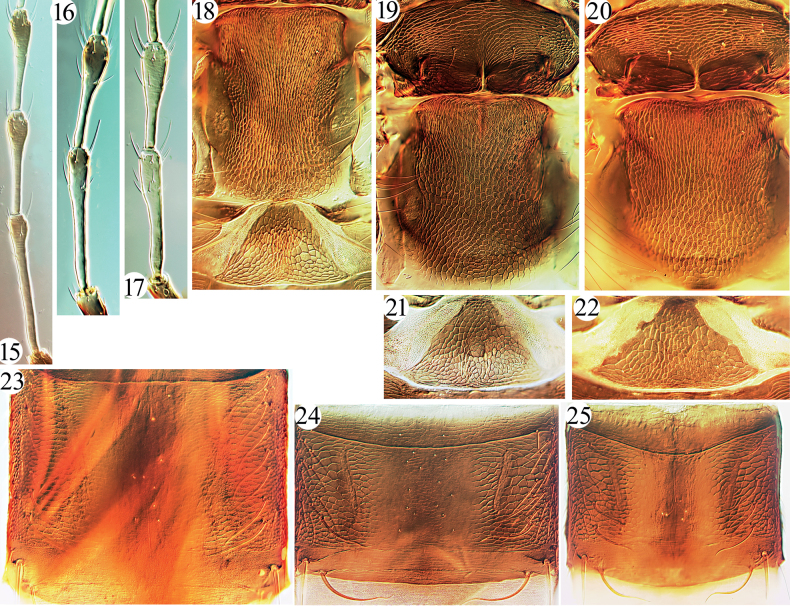
*Gigantothrips* spp. Antennal segments III–IV (**15–17**) **15***G.elegans***16***G.tibetanus* sp. nov. **17***G.yunnanensis* sp. nov.; metanotum and pelta (**18**) **18***G.elegans*; meso and metanotum (**19, 20**) **19***G.tibetanus* sp. nov. **20***G.yunnanensis* sp. nov.; pelta (**21, 22**) **21***G.tibetanus* sp. nov. **22***G.yunnanensis* sp. nov.; tegite IV (**23, 24**) **23***G.nigrodentatus***24***G.tibetanus* sp. nov.; tegite III (**25**) **25***G.elegans*.

***Thorax*.** Pronotum sculptured with irregular striae and reticulation, notopleural suture complete; two pairs of pronotal setae well developed, blunt at apex, am, aa and pa small, ml stout and elongate, epim the longest (Fig. 13); mesonotum and metanotum reticulation with internal fine markings, a pair of relatively big setae located in front half of metanotum, three tiny setae around anterior angle (Fig. 20), mesopresternum boat-shaped, metathoracic sternopleural sutures absent. All legs slender, fore tarsal absent. Fore wings broad, with about 23 duplicated cilia, sub-basal setae almost equal length, slightly blunt at apex.

***Abdomen*.** Pelta triangle and reticulate with internal markings, with a pair of campaniform sensilla and 1 pair of minute setae (Fig. 22); abdominal tergites II–VI with two pairs of major sigmoid wing-retaining setae, and 4–8 accessory slightly curved or straight setae; VII with about 8 small accessory setae laterally; S1–S3 on tergite IX short and pointed at apex, about 0.2 times as long as tube (Fig. 33); tube elongate with fine setae on the surface, 1.8 times as long as head, about 7.1 times as long as basal width, anal setae much shorter than tube; sternites II–VIII with some small setae not in lines.

**Figures 26–33.**
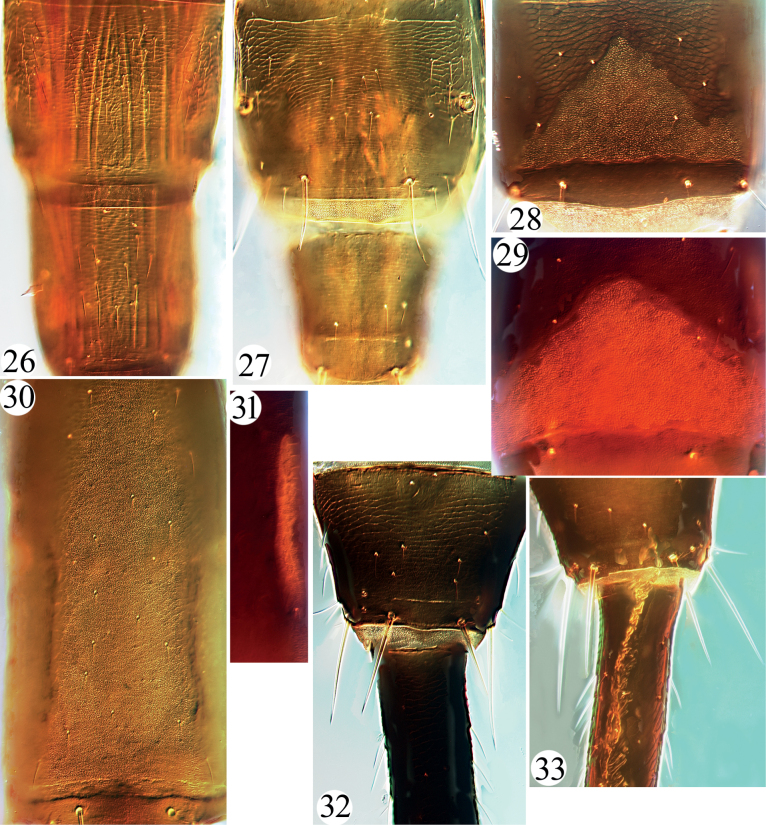
*Gigantothrips* spp. Tergites VIII–IX (**26, 27**) **26***G.pontis***27***G.xynos*; pore plate on male sternite VIII (**28–30**) **28***G.yunnanensis* sp. nov. **29***G.tibetanus* sp. nov. **30***G.nigrodentatus*; right side of male sternite VII (**31**) **31***G.tibetanus* sp. nov.; tergite IX and tube (**32, 33**) **32***G.tibetanus* sp. nov. **33***G.yunnanensis* sp. nov.

***Measurements*** (holotype female in microns). Body length 4850. Head length 430, width just behind eyes 235; eye length 120, postocular setae length S1 25, S2 30; postocellar setae length 35. Antenna length 1040, segments I–VIII length (widest) 60(50), 60(40), 215(40), 180(45), 170(45), 135(40), 100(30) and 70(15), sensoria on segment III length 60. Pronotum length 250, width 400, length of pronotal setae, am 20, aa 20, ml 40, epim 90, pa 25. Fore wing length 1700, sub-basal setae length, S1 80, S2 75, S3 100. Pelta length 150, basal width 290; tergite IX posteromarginal setae S1–S3, 155, 175, 130; tube length 780, basal width 110, apical width 60; anal setae length 195.

***Male macroptera*.** Similar to female; but smaller, fore tarsal tooth absent; abdominal tergite IX setae S2 short; sternite VIII with a pore plate, its anterior margin like ‘W’ shape and posterior margin straight and before posterior marginal setae (Fig. 28), sternites III–VII without worm-shape pore plate.

***Measurements*** (paratype male in microns). Body length 4610. Head length 415, width just behind eyes 195; eye length 125, postocular setae length S1 50, S2 35; postocellar setae length 40. Antenna length 1000, segments I–VIII length (widest) 60(50), 60(35), 210(35), 170(40), 170(40), 150(35), 95(25) and 70(15), sensoria on segment III length 55. Pronotum length 230, width 380, length of pronotal setae, am 25, aa 30, ml 70, epim 90, pa 30. Fore wing length 1600, sub-basal setae length, S1 70, S2 80, S3 60. Pelta length 135, basal width 215; tergite IX posteromarginal setae S1–S3, 150, 70, 140; tube length 740, basal width 110, apical width 55; anal setae length 200.

#### Etymology.

This species name is composed of one Latin word based on the location of type specimens.

#### Comments.

This new species was collected from leaves of *Ficustikoua*, and it is similar in body colouration to *G.nigrodentatus* from *Planchoniavalida*. It is also similar to *G.tibetanus* sp. nov. in body shape and pore plate on sternite VIII of male (Figs 28–29), but can be differentiated from both of them by having only two sense cones on antennal segment IV (Fig. 17). It is distinguished from *G.nigrodentatus* by the smaller body size, slender and yellow major body setae, and two pairs of major sigmoid wing-retaining setae on tergites II–V (in *G.nigrodentatus* large body size, major body setae stout and hyaline, and at least six pairs of major sigmoid wing-retaining setae on tergites II–V (Fig. 17)). In contrast to *G.tibetanus* sp. nov. the mid and hind tibiae are bicolored, antennal segments III–V uniformly yellow, and fore wings pale (in *G.tibetanus* sp. nov. the mid and hind tibiae uniformly brown, antennal segments III–V yellow but shaded brown at apices, and fore wings strongly shaded brown).

## Supplementary Material

XML Treatment for Gigantothrips

XML Treatment for Gigantothrips elegans

XML Treatment for Gigantothrips gallicola

XML Treatment for Gigantothrips nigrodentatus

XML Treatment for Gigantothrips pontis

XML Treatment for Gigantothrips tibetanus

XML Treatment for Gigantothrips tibialis

XML Treatment for Gigantothrips xynos

XML Treatment for Gigantothrips yunnanensis

## ﻿Additional information

### Author ORCIDs

Lihong Dang https://orcid.org/0000-0002-7571-8426

Laurence Mound https://orcid.org/0000-0002-6019-4762

Hongrui Zhang https://orcid.org/0000-0002-0089-1099

### Data availability

All of the data that support the findings of this study are available in the main text.
